# Advanced Protocol for Molecular Characterization of Viral Genome in Fission Yeast (*Schizosaccharomyces pombe*)

**DOI:** 10.3390/pathogens13070566

**Published:** 2024-07-04

**Authors:** Jiantao Zhang, Zsigmond Benko, Chenyu Zhang, Richard Y. Zhao

**Affiliations:** 1 Department of Pathology, School of Medicine, University of Maryland, Baltimore, MD 21201, USA; jiantao.zhang@som.umaryland.edu (J.Z.); chenyu.zhang@som.umaryland.edu (C.Z.); 2 Department of Molecular Biotechnology and Microbiology, Faculty of Science and Technology, University of Debrecen, 4032 Debrecen, Hungary; benko.zsigmond@science.unideb.hu; 3 Department of Microbiology-Immunology, School of Medicine, University of Maryland, Baltimore, MD 21201, USA; 4 Institute of Human Virology, School of Medicine, University of Maryland, Baltimore, MD 21201, USA; 5 Institute of Global Health, School of Medicine, University of Maryland, Baltimore, MD 21201, USA; 6 Research & Development Service, VA Maryland Health Care System, Baltimore, MD 21201, USA

**Keywords:** fission yeast, *Schizosaccharomyces pombe*, subcellular protein localization, cell proliferation, cell cycle profiling, cellular oxidative stress, autophagy, cell viability

## Abstract

Fission yeast, a single-cell eukaryotic organism, shares many fundamental cellular processes with higher eukaryotes, including gene transcription and regulation, cell cycle regulation, vesicular transport and membrane trafficking, and cell death resulting from the cellular stress response. As a result, fission yeast has proven to be a versatile model organism for studying human physiology and diseases such as cell cycle dysregulation and cancer, as well as autophagy and neurodegenerative diseases like Alzheimer’s, Parkinson’s, and Huntington’s diseases. Given that viruses are obligate intracellular parasites that rely on host cellular machinery to replicate and produce, fission yeast could serve as a surrogate to identify viral proteins that affect host cellular processes. This approach could facilitate the study of virus–host interactions and help identify potential viral targets for antiviral therapy. Using fission yeast for functional characterization of viral genomes offers several advantages, including a well-characterized and haploid genome, robustness, cost-effectiveness, ease of maintenance, and rapid doubling time. Therefore, fission yeast emerges as a valuable surrogate system for rapid and comprehensive functional characterization of viral proteins, aiding in the identification of therapeutic antiviral targets or viral proteins that impact highly conserved host cellular functions with significant virologic implications. Importantly, this approach has a proven track record of success in studying various human and plant viruses. In this protocol, we present a streamlined and scalable molecular cloning strategy tailored for genome-wide and comprehensive functional characterization of viral proteins in fission yeast.

## 1. Introduction

Fission yeast (*Schizosaccharomyces pombe*) is a single-cell eukaryotic organism that has been extensively used as a model organism to study human physiology and diseases such as cell cycle dysregulation and cancer, as well as autophagy and neurodegenerative diseases like Alzheimer’s, Parkinson’s, and Huntington’s diseases [1,2,3,4]. Given that viruses are obligate intracellular parasites that rely on host cellular machinery to replicate and produce, and fission yeast shares many of the fundamental cellular processes with higher eukaryotes, it can be utilized as a surrogate to identify viral proteins affecting these conserved cellular processes. Therefore, this approach facilitates rapid and comprehensive functional characterization of viral proteins, aiding in the identification of therapeutic antiviral targets or viral proteins with significant implications in virology [5,6]. Indeed, fission yeast has been extensively used as a model organism to study various aspects of human virology [7,8,9,10].

The protocol presented here introduces a streamlined approach for rapid and genome-wide molecular characterization of viral proteins in fission yeast [5,11,12]. It utilizes a fission yeast vector system designed for large-scale molecular cloning, allowing for gene cloning in a unidirectional fashion with positive identification of gene insertions based on α-complementation of X-gal in *Escherichia coli* (*E. coli*). The system employs an inducible gene transcriptional no message in the thiamine (*nmt*1) promoter [11,13] for gene expression, enabling the measurement of gene-specific effects. The molecular cloning process involves generating fluorescent protein (FP)-tagged viral proteins for subcellular localization determination and functional characterization of viral proteins without FP tags. Different strengths of the *nmt*1 promoter and various cell growth selection markers allow for the testing of gene expression at different levels [11,13]. Additionally, different fluorescent proteins, such as green fluorescent protein (GFP), yellow fluorescent protein (YFP), and cyan fluorescent protein (CFP), can be used for observing FP-fused proteins and conducting co-localization tests. Fission yeast cells transformed with viral-gene-carrying plasmids can be selected simultaneously on agar plates, facilitating rapid testing of multiple viral genes. Furthermore, the cells are easy to maintain with a relatively short doubling time, allowing for simultaneous measurement of viral-gene-specific and gain-of-function activities under the same inducible conditions. Importantly, this protocol has been successfully applied to conduct genome-wide studies of various human and plant viral genomes, including human immunodeficiency virus type 1 (HIV-1), barley yellow dwarf virus (BYDV), Zika virus (ZIKV), and SARS-CoV-2 [14,15,16,17].

**Figure 1:**
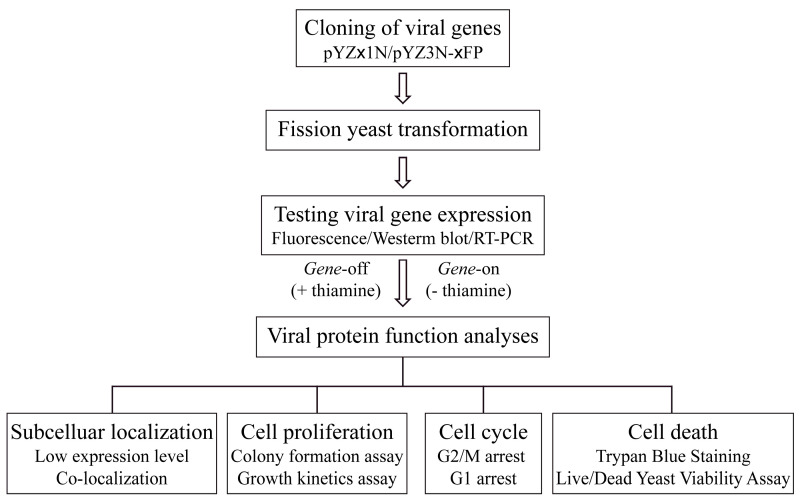
Flow chart illustrating the workflow for studying viral proteins in fission yeast.

In this protocol, we present a streamlined and scalable molecular cloning strategy tailored for genome-wide and comprehensive functional characterization of viral proteins in fission yeast (Figure 1). Specific experimental procedures and examples, from published or unpublished data, are provided for determining subcellular locations of cloned viral proteins and characterizing their effects on basic cellular activities such as cell proliferation, cell cycle regulation, cellular oxidative stress, autophagy, and cell viability. Please note that this protocol is an updated version based on a previously published protocol in a book chapter with newly added information and unpublished data [12]. For further information on using fission yeast to study virus–host interactions involving highly conserved viral proteins, refer to relevant literature reviews [5,9,18,19].

## 2. Materials

### 2.1. Fission Yeast Strains, Plasmids, and Molecular Cloning

See Table 1 for details.

The described fission yeast gene expression vector system here utilizes the pYZ vector series [11], which is derived from the pREP series [13,22]. These modified vectors are designed to enable easy identification of cloned gene insertions and fusion with the xFP fluorescent gene, with ‘x’ representing different colors of FPs, for in vivo gene expression analysis. Alongside the previously published pYZx3N-GFP vector [11], our laboratory has also constructed pYZx3N-YFP and pYZx3N-CFP (unpublished data). The pYZx1N vectors are employed for assessing viral gene functions, while the pYZ3N-xFP vectors are utilized to determine the intracellular localization of viral proteins by tagging them with an xFP at the N-terminal end of the target protein. All these plasmids feature an inducible *nmt*1 promoter of varying strengths [13,22]. The pYZ1N or pYZ3N vector series includes a *LEU*2 gene as the selection marker, while pYZ2N-related vectors carry a *URA*4 gene for selection. Additionally, we have developed a pYZ1N-*bleMX*6 plasmid employing Zeocin for selection (see Table 1). For more options of gene cloning or tagging vectors, refer to [23,24].

To carry out gene cloning of viral genes in fission yeast and verification by methods like restriction enzyme analysis, the following materials are needed in addition to fission yeast gene cloning plasmids as listed in Table 1 (also refer to [11]).

**Figure 2:**
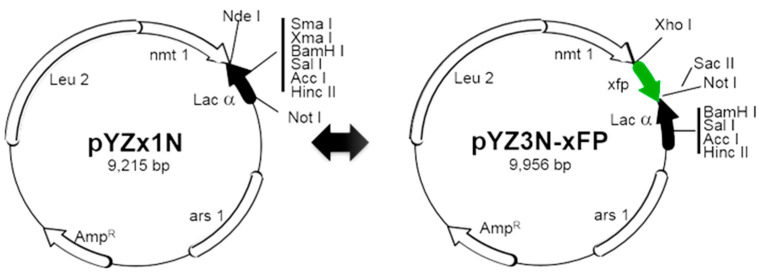
A schematic diagram detailing a shotgun approach for cloning a viral genome in fission yeast. On the left side, the pYZx1N vector series is depicted, comprising three gene expression plasmids denoted as pYZ1N, pYZ41N, and pYZ81N, where x can be 1, 4, or 8. pYZ1N contains the wild-type *nmt*1 promoter, while pYZ41N and pYZ81N contain attenuated *nmt*1 promoters with TATA box mutations. All three vectors carry the *LEU*2 gene, whereas pYZ2N carries the wild-type *nmt*1 promoter and a *URA*4 gene selection marker. On the right side, the pYZ3N-xFP construct is presented, featuring the wild-type *nmt*1 promoter and fluorescent protein xFP [28,29,30], with x representing GFP, YFP, or CFP. Selection of DNA insert with α-complementation utilizes the α-peptide of β-galactosidase. Unique cloning sites within these vectors are highlighted. Other components of the plasmid include *ars*1, the origin of replication from *S. pombe*; *LEU*2, the *Saccharomyces cerevisiae* leucine biosynthesis gene; and *Amp*R, the bacterial ampicillin resistance gene. This depiction is adapted from [11]. Note: There are multiple *Acc*I and *Hinc*II sites on both plasmids. Thus, these sites are not single restriction cutter sites. Also, note that this figure has previously been included in a book chapter [12]. As per our contractual obligations with the publisher, we retain the rights to republish this material, and the relevant copyright information is reproduced verbatim.

1. PCR primers specific to the viral gene for amplification;
2. PCR kit containing DNA polymerase and nucleotides (dNTPs);
3. DNA gel extraction kit for purification of PCR products;
4. Restriction enzymes and respective buffers specific to the cloning strategy (e.g., for linearizing the plasmid vector and digesting the insert, see Figure 2);
5. DNA ligase for joining viral gene inserts with plasmid DNA;
6. Gel electrophoresis apparatus for analyzing DNA fragments;
7. DNA ladder or marker for size determination;
8. Agarose gel and TAE or TBE buffer for gel electrophoresis;
9. UV transilluminator or an imaging system for visualizing DNA bands;
10. A wild-type fission yeast strain such as SP223 or 972 for plasmid DNA transformation;
11. Selective media for fission yeast (e.g., minimal medium supplemented with appropriate nutrients; also refer to Section 2.2. Fission Yeast Growth Media).

### 2.2. Fission Yeast Growth Media

Prepare all solutions using deionized water and analytical-grade reagents. Ensure that all reagents are prepared and stored at room temperature unless specified otherwise.

1. Standard Yeast Extract with Supplements (YES) medium: 0.5% (*w*/*v*) yeast extract, 3.0% (*w*/*v*) glucose, supplements: 225 mg/L adenine (Ade), leucine (Leu) and uracil (Ura) (see Note 1).
2. Edinburgh Minimal Medium (EMM): 14.7 mM potassium hydrogen phthalate, 15.5 mM Na_2_HPO_4_, 93.5 mM NH_4_Cl, 2.0% (*w*/*v*) glucose, 20 mL/L 50× salt stock, 1 mL/L 1000× vitamin stock, 0.1 mL/L 10,000× mineral stock (see Note 2).
3. 50× Salt Stock: 0.26 M MgCl_2_, 5 mM CaCl_2_, 0.67 M KCl, 14.1 mM Na_2_SO_4_.
4. 1000× Vitamin Stock: 4.20 mM pantothenic acid, 81.2 mM nicotinic acid, 55.5 mM inositol, 40.8 μM biotin.
5. 10,000× Mineral Stock: 80.9 mM boric acid, 23.7 mM MnSO_4_, 13.9 mM ZnSO_4_∙7H_2_O, 7.40 mM FeCl_2_∙6H_2_O, 2.47 mM molybdic acid, 6.02 mM KI, 1.60 mM CuSO_4_∙5H_2_O, 47.6 mM citric acid.
6. Pombe Minimal Glutamate (PMG) Medium: potassium hydrogen phthalate 3 g/L (14.7 mM); Na_2_HPO_4_ 2.2 g/L (15.5 mM); L-glutamic acid, monosodium salt (3.75 g/L); glucose 20 g/L (2% *w*/*v*); salt mix 20 mL/L; vitamin mix 1 mL/L; mineral mix 0.1 mL/L (see Note 3).
7. For solid fission yeast media, add 2% (*w*/*v*) agarose.
8. Luria–Bertani (LB) Medium: 1% (*w*/*v*) tryptone, 0.5% (*w*/*v*) yeast extract, 1% (*w*/*v*) NaCl (see Note 4).
9. For solid LB media, add 1.5% (*w*/*v*) agarose.

### 2.3. Inducible Gene Expression in Fission Yeast

To induce expression of a viral gene of interest in fission yeast, use the same EMM or PMG media as listed in Section 2.2. Additionally, prepare a 20 mM stock solution of thiamine and sterilize it by using an autoclave. This solution will be utilized to modulate the expression of the viral gene under the control of the *nmt*1 promoter, described as follows.

In this inducible gene expression system, viral gene expression can be suppressed or induced in the presence or absence of thiamine, respectively [13,22]. The pYZ1N vector contains the wild-type *nmt*1 promoter, facilitating high-level viral gene transcription. Conversely, pYZ41N and pYZ81N carry attenuated *nmt*1 promoters with mutations in the TATA box, resulting in intermediate (pYZ41N) and low (pYZ81N) levels of gene transcriptional activity [11,13,22]. Full gene induction is achieved by removing thiamine from the growth medium through vigorous washes with water. Conversely, the addition of 20 µM thiamine effectively suppresses gene transcription. However, even with thiamine in the growth medium suppressing gene expression, there is always a low level of gene expression depending on which version of the *nmt*1 promoter is used [25]. Therefore, it is crucial to determine the gene expression level under gene-inducing conditions relative to gene-suppressing conditions. The difference in gene expression levels between gene-inducing and gene-repressing conditions is approximately 300-, 24-, or 6-fold when the *nmt*1, *nmt*41, or *nmt*81 promoter is used, respectively [25]. Since the level of thiamine in the growth medium regulates *nmt*1-driven gene expression [22], the level of gene expression under the same *nmt* promoter can be lowered by adding various levels of thiamine in the range of 10–50 nM thiamine [26]. Similarly, a low level of thiamine has been used to minimize the basal level of gene expression [27]. Typically, upon thiamine removal from the growth medium, initial gene transcription is observed at approximately 10 h, with full transcriptional activity achieved by about 16 h [13].

### 2.4. Determination of Subcellular Localization

The materials needed are as follows:

1. Microscope slides and coverslips.
2. DAPI staining stock solution in water (1 mg/mL). Store at −20 °C. Use 1 µg/mL in the final concentration.
3. Hoechst 33342 staining stock solution in water (1 mg/mL). Store at −20 °C. Use 1 µg/mL in the final concentration.
4. Sodium citrate (pH 7.0) stock solution (500 mM). Store at room temperature. Use 50 mM in the final concentration.
5. Propidium iodine (PI) stock solution in water (4 mg/mL). Store in the dark at −20 °C. Use 0.1 mg/mL in the final concentration.
6. RNase A (10 mg/mL; boil 10 min, cool to RT, filter and store at −20 °C). Use 0.1 mg/mL in the final concentration.
7. Trypan Blue solution (0.4% (*w*/*v*)). Store at room temperature. Use 0.2% (*w*/*v*) in the final concentration.
8. NH_4_Cl (1.4 M). Store at room temperature.
9. Thiamine stock solution in distilled water (20 mM). Store at −20 °C.
10. GH solution (2% (*w*/*v*) D-(+)-glucose +10 mM Na-HEPES, pH 7.2). Store at room temperature.
11. FUN-1 working solution (80 μM). Store at −20 °C [27].
12. Leica DM fluorescent microscope with 11001v2 long-path Chroma filter cube.

### 2.5. Western Blot Analysis of Fission Yeast Proteins

The essential materials needed to carry out Western blotting analysis are the following:

1. Protein samples from fission yeast (cell lysates or purified proteins).
2. SDS-PAGE gel or other appropriate gel system for protein separation.
3. Transfer buffer suitable for fission yeast proteins (e.g., tris-glycine buffer).
4. Nitrocellulose or PVDF membrane.
5. Transfer apparatus (e.g., wet or semi-dry transfer system).
6. Blocking buffer compatible with fission yeast proteins (e.g., 5% (*w*/*v*) non-fat dry milk or BSA in PBS-T).
7. Primary antibody specific to the fission yeast protein of interest.
8. Secondary antibody conjugated with HRP (horseradish peroxidase) compatible with fission yeast proteins.
9. Chemiluminescent substrate suitable for detection of fission yeast proteins (e.g., ECL).
10. Film or chemiluminescent imaging system.
11. Washing buffer compatible with fission yeast proteins (e.g., PBS-T).
12. Electrophoresis apparatus suitable for running SDS-PAGE gels.
13. Protein marker or ladder.
14. Blocking agent (e.g., non-fat dry milk or BSA).
15. Gel documentation system or imaging equipment for visualization and analysis.

Note that additional materials or specific variations may be needed depending on the experimental setup.

## 3. Methods

### 3.1. Genome-Wide Molecular Cloning of Viral Proteins

To clone multiple viral genes simultaneously, a large-scale cloning fission yeast gene expression vector system is employed, as previously outlined (Figure 2) [11,12]. The specific molecular cloning procedures are detailed below, utilizing pYZ1N and pYN3N-GFP as examples:

1. Viral genes are inserted into the destination vector using the traditional cloning method, which involves the use of restriction enzymes to cut DNA and ligase to join DNA. For instance, to clone a viral gene into the pYZ1N plasmid, first amplify the gene via PCR with a forward primer containing an *NdeI* restriction site and a reverse primer containing the stop codon, along with one of the following single-cutter restriction sites: *NdeI*, *XmaI*, *XmaI*, *BamHI*, *SalI*, or *NotI* on the pYZ1N plasmid (Figure S1). After amplification, ligate the digested PCR products into the vector following the same digestion. Note that the *NdeI* site already includes a start codon (ATG) and using the *NotI* site will remove the entire Lac fragment preceding the Nmt1 terminator sequence (see Note 11).
2. To clone a C-terminus GFP-tagged viral gene into the pYZ3N-GFP, utilize the single-cutter *XhoI* site (Figure S2). The forward primer should include the start codon, while the reverse primer should remove the stop codon. Note the presence of an in-frame stop codon at the end of GFP immediately following the *SacII*/*NotI* sites.
3. To clone an N-terminus GFP-tagged viral gene into the pY3N-GFP, employ the single-cutter *SacII* or *NotI* restriction enzyme (Figure S2). Employing *SacII*/*NotI* and *XmaI*/*XmaI* restriction enzymes for viral gene insertion will result in the removal of the full Lac fragment from the pYZ3N-GFP plasmid (Figure S2). This ensures the proper orientation of the N-terminus tagged viral gene. In this scenario, the stop side primer must also contain an in-frame stop codon.
4. To clone a viral gene fused with GFP directly into the pYZx1N plasmid, overlapping PCR or three-fragment ligation could be employed. Amplify the viral gene and xFP separately via PCR, using viral genome for the viral gene and any DNA source for xFP amplification. Similarly to Section 1, utilize the combination of *NdeI* with *NdeI*, *XmaI*, *XmaI*, *BamHI*, *SalI*, or *NotI* (Figure S1).
5. Recent advances in molecular cloning methodologies utilize other properties of DNA polymerase, such as exonuclease activity, along with DNA homologous recombination to simplify the cloning process could also be used. Techniques like NEBuilder HiFi DNA Assembly, Gibson Assembly [31], and Takara in-fusion cloning can be employed to create these constructs. Taking NEBuilder HiFi DNA Assembly as an example, to clone a C-terminus xFP-tagged viral gene into the pYZ1N plasmid, amplify the viral gene using a forward primer (VG-F) containing a 5′-end overlapping sequence of 20–30 bp with the *nmt* promoter of the pYZ1N plasmid and a 3′-end overlapping sequence of 20 bp with the viral gene start side (containing the ATG start codon), and a reverse primer (VG-R) containing a 5′-end overlapping sequence of 15–30 bp with the xFP start side and a 3′-end overlapping sequence of 20 bp with the viral gene end side (excluding the stop codon). Amplify xFP using a forward primer (xFP-F) containing a 5′-end overlapping sequence of 15–30 bp with the viral gene end part (excluding the stop codon) and a 3′-end overlapping sequence of 20 bp with the xFP gene start side, and a reverse primer (xFP-R) containing a 5′-end overlapping sequence of 20–30 bp with the *nmt* terminator of the pYZ1N plasmid and a 3′-end overlapping sequence of 20 bp with the xFP end side (including the stop codon). After digesting pYZ1N with *NdeI*-*NotI*, the three fragments (digested plasmid plus two PCR products) can be assembled easily using the “NEBuilder^®^ HiFi DNA Assembly” kit in one step. See the flanking sequences of pYZ1N in Figure S1.
6. Verification of the insert and its orientation is crucial. Firstly, this can be achieved by PCR or restriction digestion using one of the described cloning methods and an inner restriction site if a single restriction site was used for insertion. Subsequently, the final plasmid should be further confirmed by Sanger sequencing or whole plasmid sequencing.
7. Carefully design primers for gene amplification to ensure that the viral gene and/or the xFP ORF are in frame.
8. This flexible and reverse order of the cloning strategy has proven highly beneficial and has been successfully applied in our laboratory for the shotgun cloning of HIV-1, BYDV, ZIKV, and SARS-CoV-2 genomes, respectively [14,15,16,17].

### 3.2. Recombinant DNA Transformation and Inducible Viral Gene Expression

1. The resulting pYZ plasmids carrying viral genes are introduced into the wild-type fission yeast SP223 strain via electroporation [11,15], followed by maintenance in selective EMMs supplemented with 20 µM of thiamine to inhibit viral gene expression.
2. Transformants are selected based on either *Leu* or *Ura* auxotrophy on selective EMM, depending on whether the plasmid carries a *LEU*2 or *URA*4 gene.
3. Successful transformation of the respective viral-gene-containing plasmid is confirmed through single-colony PCR [15].
4. Conditional induction of viral gene expression is achieved by thiamine removal from the growth medium, facilitating the measurement of viral-gene-specific activities over time [13,22]. Importantly, all viral activities can be concurrently measured under identical inducible conditions, expediting the functional characterization of the viral genome of interest.
5. Verification of viral gene expression can be accomplished in the following ways:
  - Observing xFP production using a fluorescent microscope;
  - Measuring viral protein production by Western blot analysis with a monoclonal antibody;
  - Measuring mRNA levels using quantitative RT-PCR.
6. To assess viral-gene-specific activities, a single yeast colony containing a specific viral-gene-containing plasmid is cultured to the logarithmic (log) phase in special liquid EMM supplemented with 20 µM of thiamine.
7. Cells are subsequently harvested and washed three times with distilled water to eliminate thiamine.
8. Finally, 2 × 10^5^ cells/mL, or a cell concentration that is determined empirically for optimal gene expression, are re-inoculated into fresh, specific liquid EMM without thiamine to induce gene expression (Gene-on) or with thiamine to suppress gene expression (Gene-off), which serves as the control (see Notes 5 and 6).
9. The cell suspensions are then incubated at 30 °C with constant shaking (300 rpm) before observation [13,22].

### 3.3. Determination of Subcellular Localization of Viral Proteins

**Figure 3:**
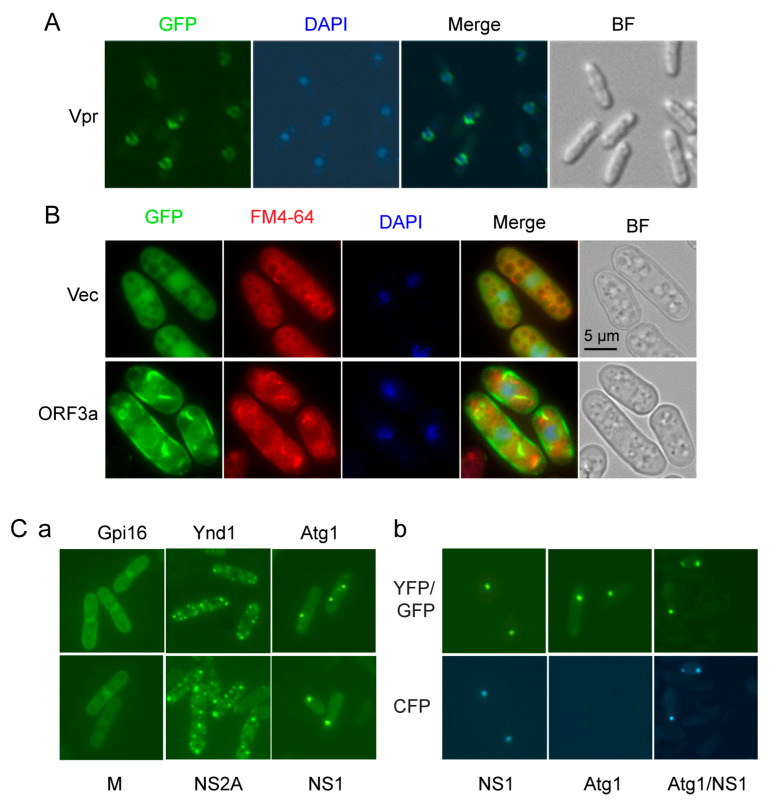
Determining the intracellular localization of viral proteins. All GFP-viral proteins were produced from the pYZ3N-GFP gene expression vector under conditions of low gene expression and observed within 20 h of gene induction using fluorescence microscopy. (**A**) Co-localization of an HIV-1 viral protein, R (Vpr), on the nuclear membrane, with nuclei stained using DAPI. In this panel, “BR” represents the bright field, “GFP” indicates the localization of the GFP-Vpr fusion protein, “DAPI” shows the same cells stained with DAPI, and “Merge” displays the merged picture of GFP and DAPI to demonstrate co-localization. (**B**) Association of SARS-CoV-2 ORF3a protein with lysosomal vacuoles as shown in mammalian cells [34,36]. Vec, vector control; BF, bright field; FM4-64, a lysosomal vacuole-specific dye (our unpublished data) [37]. (**C**) Comparison of ZIKV viral protein localization with cellular proteins known to localize in the endoplasmic reticulum (ER) (Gpi16-YFP), Golgi (Ynd1-YFP), and cytoplasmic puncta (Atg1-YFP), an indication of cellular autophagy induction [14] (**a**). All proteins shown here are tagged with GFP [14]. (**b**) Co-localization of GFP-NS1 with Atg1-YFP showing cellular autophagy-induced cytoplasmic puncta under nitrogen starvation. Note that both GFP and YFP were detectable under the GFP/YFP filter. However, only the GFP signal was seen under the CFP filter. Arrow indicates where Atg1-YFP was co-located with GFP-NS1. Note that part of this figure has previously been included in a book chapter [12]. As per our contractual obligations with the publisher, we retain the rights to republish this material, and the relevant copyright information is reproduced verbatim.

1. The viral proteins fused to GFP are expressed as described above, and their subcellular locations are determined using fluorescent microscopy. To mitigate artifacts stemming from high-level expression of the viral proteins, 10 nM of thiamine is added to the EMM to reduce the level of viral protein expression [12,32] (refer to Note 6, 7). Typically, the subcellular localization of each viral protein is visualized within 20 h after gene induction using fluorescent microscopy.
2. To aid in determining the subcellular location of a protein, a fluorescent DNA dye, 4′,6′ diamino-2-phenylindole (DAPI) for fixed cells or Hoechst 33342 for live or fixed cells, is commonly used to stain nuclei, enabling differentiation of whether the viral protein is associated with the nucleus or other subcellular compartments.
3. For Hoechst 33342 or DAPI staining, 2–5 µL of the viral-gene-expressing cell suspension is pipetted onto a glass slide. The cells are heat-fixed for 1 min at 70 °C on a hot plate. The slide is then cooled for a few seconds before adding Hoechst 33342 or DAPI (1 µg/mL). A coverslip is placed over the cells. Cells expressing GFP-fused viral proteins and stained with Hoechst 33342 or DAPI are observed under fluorescence microscopy (refer to Figure 3).
4. To validate the specific subcellular location of a viral protein, fission yeast cellular proteins known to localize specifically in, for example, nuclear membrane localization of HIV-1 Vpr protein (Figure 3A) [33], lysosomal localization of SARS-CoV-2 ORF3a protein (Figure 3B) [16,34], and various ZIKV proteins localized in the endoplasmic reticulum (Gpi16), Golgi (Ynd1), and mitochondria (Rsm10) [14] can be utilized for comparison (Figure 3Ca) (refer to Note 8).
5. Additionally, the induction of cellular autophagy by a viral protein can be detected by the formation of autophagy-related puncta structures in the cytoplasm using one of many autophagy-related Atg protein markers, such as Atg1 (Figure 3Cb) [35].
6. Image analysis is conducted using a Leica DMR fluorescence microscope equipped with a high-performance charge-coupled device camera (Hamamatsu) and Open-Lab software (Improvision, Inc., Lexington, MA, USA). For GFP observation, a Leica L5 filter with excitatiominn at 480/40 nm and emission at 527/30 nm is used. A Leica YFP filter with excitation at 500/20 nm and emission at 535/30 nm is employed for YFP observation. Note that the spectrum of GFP and YFP overlaps. Therefore, we cannot distinguish these two proteins under the GFP/YFP filter. However, we could use the deduction method to detect the GFP signal under the CFP filter (Figure 3Bb). For CFP observation, a specific Leica CFP filter with excitation at 436/20 nm and emission at 480/40 nm is utilized (refer to **Note 9**).

### 3.4. Measurement of Cell Proliferation

**Figure 4:**
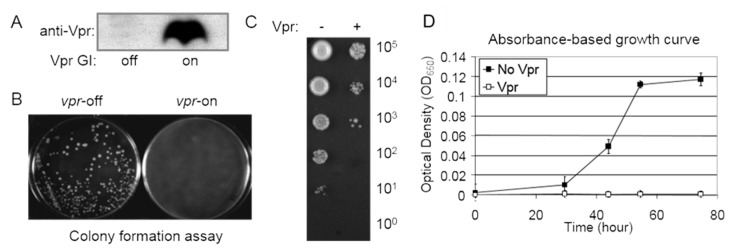
Measurement of cell proliferation using different experimental assays. (**A**) Western blot analysis demonstrating the inducible expression of the HIV-1 *vpr* gene [33]. (**B**) Colony formation assay exhibiting the production of HIV-1 Vpr protein in fission yeast, which inhibits colony formation [33]. (**C**) Semi-quantitative colony-forming assay utilized to demonstrate how Vpr prevents colony formation on an agar plate. Cells with Vpr turned off (-) or on (+) were cultivated in liquid EMM and collected 48 h after gene induction. A 10-fold series of dilutions were plated on a selective EMM agar plate. (**D**) Absorbance-based growth kinetics determination of the Vpr effect on cellular growth. “No Vpr” refers to cells without *vpr* gene expression, grown in a repressing liquid media, while “Vpr” culture indicates cells in which *vpr* is expressed, resulting in little or no cellular growth. Note that we omitted a protein loading control in (**A**) because the HIV-1 Vpr protein was generated under identical experimental conditions using an inducible *nmt*1 promoter. Therefore, the only difference between the *vpr* gene-producing (off) and gene-repressing (on) conditions is the presence or absence of thiamine. Note that this figure has previously been included in a book chapter [12]. As per our contractual obligations with the publisher, we retain the rights to republish this material, and the relevant copyright information is reproduced verbatim.

1. To assess whether a viral protein influences cellular proliferation, the colony formation assay is employed to gauge cell growth and viability [16,38,39], alongside the cellular growth kinetics assay to quantify cellular growth, as previously described [16,20,33].
2. A single yeast colony containing a viral-gene-containing plasmid is selected from the selective EMM plate and cultured overnight in specific liquid EMM supplemented with thiamine. The following day, 1 mL of mid-log-phase culture is centrifuged, washed three times with distilled water, and the cells are resuspended in an appropriate volume of EMM. Approximately 100 µL of liquid cultures containing around 1000 cells is spread onto selective EMM agar plates with and without thiamine. Inducible protein production can be accessed via Western blot analysis (Figure 4A). The agar plates are then incubated at 30 °C for 4–6 days to observe the presence or absence, as well as the sizes, of forming colonies. The absence of colonies on the agar plates may indicate a potential cell-killing effect, while smaller colony sizes compared to the control typically suggest potential growth restriction (Figure 4B).
3. A semi-quantitative colony assay is employed to further assess the extent of growth inhibition presumably observed in the colony formation assay described above.
4. Using the same preparation procedure as described above, approximately 1–5 µL (instead of 100 µL) of liquid EMM cultures with serial 10-fold dilutions (ranging from 10^6^ to 10^0^ cells) is spotted onto selective EMM agar plates. The plates are then incubated at 30 °C for 3–6 days to observe the level of colony formation dilution and the number of cells within each colony (at low dilutions) as a semi-quantitative indicator of the viral effect on cellular growth or cytotoxicity (see an example in Figure 4C).
5. For quantitative measurements of growth inhibition, a growth kinetics assay is employed (Figure 4D). Specifically, liquid cell cultures are grown in a 96-well microtiter plate containing 100 µL of selective EMM. Cell cultures are prepared as described above and incubated at 30 °C in a moist incubator. Cellular growth is monitored at OD_650_ over the specified time period using a spectrophotometer (Figure 4D) after shaking the cultures in the 96-well plate.

### 3.5. Cell Cycle Profiling

**Figure 5:**
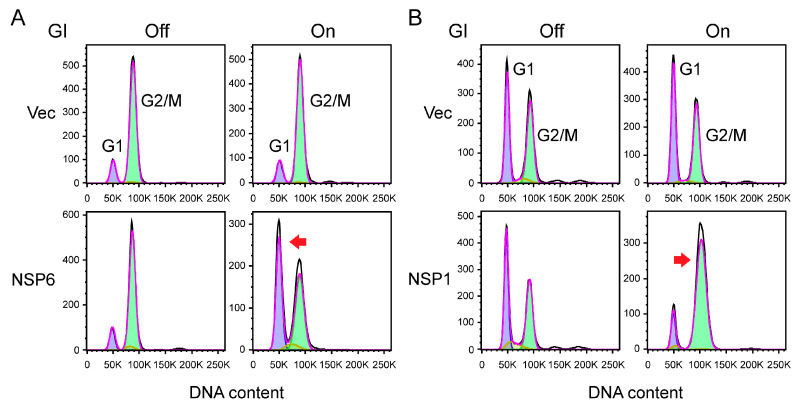
Cell cycle profiling. (**A**) Effect of SARS-CoV-2 NSP6 protein on cell cycle G1 regulation. (**B**) Effect of SARS-CoV-2 NSP1 protein on cell cycle G2/M regulation. Cell cycle profiles were assessed by analyzing DNA content using flow cytometry 48 h after gene induction. Arrows highlight significant increases or decreases in DNA content. “GI” denotes gene induction, “Off” indicates gene-suppressed conditions, and “On” represents gene-induced conditions. These are new and unpublished data generated in our laboratory.

Fission yeast is a haploid organism [29,40]. Cells in the mitotic G1 phase of the cell cycle are identified by their single copy (1N) of haploid DNA, while the G2/M phase of the cell cycle exhibits double the amount of haploid (2N) DNA. The S phase typically lies between 1N and 2N DNA due to ongoing DNA replication (refer to Figure 5). Hence, the cell cycle profile of viral protein-expressing cells can be determined by measuring the DNA content using flow cytometry, as previously outlined [14,15,33]. Specifically, the following steps are taken:

1. Cells containing the pYZ1N viral gene are cultured to the stationary phase in 5 mL of EMM containing thiamine, with constant shaking at 30 °C. A 1 mL aliquot of the culture is collected, washed three times with distilled water to eliminate thiamine, and re-inoculated into 5 mL of culture medium at a concentration of 2 × 10^5^ cells per mL with or without thiamine.
2. Cells are harvested in approximately 48 h. Spin down 10^7^ cells from the liquid culture at 2000 revolutions per minute (rpm) for 5 min, fix the cells with 1 mL of 70% cold ethanol, and store at 4 °C.
3. Before flow cytometry analysis, take 200 µL of cells and add them to 2 mL of 50 mM sodium citrate (pH 7.0) in a 5 mL Falcon tube, mix well, and centrifuge at 2000 rpm for 5 min. Treat the cells with RNase A (0.1 mg/mL) in 50 mM sodium citrate for 2 h at 37 °C, and then stain with propidium iodide (PI, 4 µg/mL) on ice for at least 1 h.
4. To test whether production of a viral protein in fission yeast induces cell cycle G1 arrest, fission yeast cells that are cultured in regular EMM can be used in flow cytometric analysis, as fission yeast cells primarily reside in the G2/M phase of the cell cycle [33,41] (refer to Figure 5A, top, our unpublished data). Here, we show that expression of SARS-CoV-2 NSP6 protein significantly shifts the cell cycle profile from the G2 phase to the G1 phase (Figure 5A, top, our unpublished data).
5. To evaluate whether viral protein production causes cell cycle G2/M arrest, fission yeast cells need to first be cultured in low-nitrogen (LN) medium containing 2.5 mM NH4Cl, which enriches fission yeast cells in the G1 phase of the cell cycle [12,33] (refer to Figure 5B, top, our unpublished data). As we show here, the expression of SARS-CoV-2 NSP1 protein significantly shifts the cell cycle profile from the G1 phase to the G2/M phase (Figure 5B, bottom, our unpublished data).
6. Both EMM and LN media can be utilized to measure the effect on the S phase.
7. The DNA content is analyzed on a FACSCanto II (Becton Dickinson) using the FACS DIVA 6.3 software (Becton Dickinson) or whatever software is available. Ten thousand events are collected, and the level of DNA content corresponding to cells in G1, G2/M, or S is determined as the FL2 parameter (FL-2 measures the amount of PI fluorescence emitted through a 585 nm band-pass filter). When PI binds to nucleic acid, it has an excitation maximum of ~535 nm and an emission maximum of ~615 nm.

### 3.6. Measurement of Cell Death

**Figure 6:**
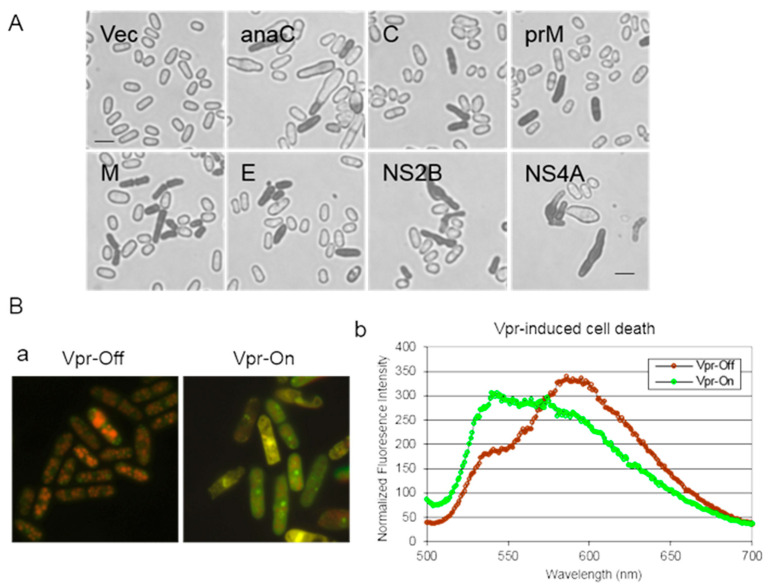
Measurement of cell death in fission yeast using different assays. (**A**) Cell death induced by various ZIKV viral proteins is assessed 48 h after gene induction through Trypan Blue staining [14]. (**B**) The Yeast Live/Dead Assay to illustrate HIV-1 Vpr-induced cell death, also measured 48 h after gene induction [27]. (**a**) Qualitative observation: viable yeast cells (Vpr-Off), which are metabolically active and possess intact plasma membranes, can convert the fluorescent substrate FUN-1 from a diffuse green color to a compact orange–red fluorescent metabolite within cells (**left**). After 48 h of Vpr induction (Vpr-On), cells fail to convert FUN-1, remaining diffuse green–yellow, indicating cell death (**right**). (**b**) Quantitative measurement, where two clearly distinguishable peaks at maximum wavelengths of 590 nm and 540 nm represent live cells and dead cells, respectively. Note that this figure has previously been included in a book chapter [12]. As per our contractual obligations with the publisher, we retain the rights to republish this material, and the relevant copyright information is reproduced verbatim.

1. Cell viability can be assessed using either Trypan Blue staining [14,42] or a commercial Live/Dead Yeast Viability Assay (Invitrogen) [27,39,43].
2. To quantify the number of dead cells, cell cultures are prepared as described above. The percentage of cell death induced by a viral protein is monitored over time following viral gene induction. Trypan Blue (Thermo Fisher Scientific, Waltham, MA, USA) at a final concentration of 0.2% (*w*/*v*) is introduced into the cell culture. Trypan Blue, a diazo dye, exclusively stains dead cells; live cells with intact membranes remain unstained. Consequently, the percentage of cell death is determined by counting the number of Trypan Blue-stained cells relative to the total number of cells counted (refer to Figure 6A).
3. Cell viability can also be assessed using a commercial Live/Dead Yeast Viability Assay (Cat. No. L-7009; Invitrogen, Carlsbad, CA, USA), adapted for fission yeast [27,39,43]. This assay measures cell viability by monitoring intracellular metabolic activity through FUN-1 staining. Metabolically active cells convert the yellow-green, fluorescent intracellular FUN-1 into red-orange intra-vacuolar structures, emitting fluorescence at 590 nm. Metabolically inert or dead cells exhibit bright, diffuse, green–yellow fluorescence at ~540 nm (Figure 6B).
4. Briefly, thiamine is removed from a log-phase cell culture as described previously. Cells are then diluted to a concentration of 5 × 10^4^ cells/mL and resuspended in EMM supplemented with or without thiamine to suppress or induce viral gene expression, respectively. The cell culture is incubated at 30 °C with constant shaking at 300 rpm and collected over time.
5. The cell culture is resuspended in GH solution (2% (*w*/*v*) D-(+)-glucose +10 mM Na-HEPES, pH 7.2). A 50 µL aliquot of FUN-1 solution (80 µM) is added to an equal volume of cell suspension. The suspension is further incubated at 30 °C for 45 min. Approximately 3 µL of the suspension is applied onto a glass slide and covered with a coverslip. Cell viability is examined using a Leica DM fluorescent microscope equipped with a 11001v2 long-path Chroma filter cube. Typically, actively respiring cells are clearly marked with orange–red fluorescent structures at a maximum wavelength of approximately 590 nm, while metabolically inert or dead cells exhibit bright, diffuse, green–yellow fluorescence at a maximum wavelength of approximately 540 nm [27,39,43]. FUN1-stained cell images are captured using red (N2.1, emission LP 590 nm) and green (YFP, emission 535/30 nm) filters. Final images are generated by merging fluorescence (refer to Figure 6B).

## 4. Notes

1. The YES medium is the most commonly used complete and rich medium and is normally used to grow fission yeast cells without selection.
2. EMM is typically used to select for the presence of a plasmid that carries either a *LEU*2 gene or *URA*4 gene in fission yeast cells that are deficient in *leu*1-32 or *ura*4-294 such as SP223. Supplement with adenine, uracil, or leucine (225 mg/L) to complement the corresponding auxotrophic mutants of the yeast strain. Normally, 20 μM of thiamine is used to suppress the *nmt*1 promoter.
3. As only a limited number of auxotrophic nutrient deficiency markers are available for selection, antibiotic resistance markers are good alternatives. However, antibiotic resistance markers typically work well in complete rich medium but not in defined EMM. We found that Zeocin works well in PMB medium to select and maintain *bleMX*6 resistance in conjunction with a *LEU*2 auxotrophic marker in fission yeast [21].
4. Supplement LB medium with ampicillin (100 μg/mL) to select for the presence of the plasmid. This medium is used for growing bacterium *E. coli* Top 10 or DH5α cells and for plasmid DNA transformation.
5. The unique features of this vector system are as follows: It simplifies the gene cloning process into two fission yeast pYZ vectors in a sequential manner. All gene cloning occurs unidirectionally, ensuring positive identification of gene insertions. An inducible gene transcriptional *nmt*1 promoter [11,13,22] is utilized to facilitate the determination of gene-specific effects. Three different strengths of the *nmt*1 promoter (high, intermediate, and low) are coupled with two distinct cell growth markers (*leu*2 and *ura*4) and one antibiotic selection marker (bleMX6) [11,13,21,22], enabling the testing of the gene expression at various levels. Three different fluorescent proteins (GFP, YFP, and CYP) are available for evaluating subcellular localization and co-localization of viral proteins within cells.
6. Protein overproduction in fission yeast cells can lead to the formation of protein aggregates, potentially obscuring the true subcellular location of the protein of interest. To mitigate this artifact, 10 nM of thiamine is added to the EMM to reduce viral protein expression levels [15,44]. This precaution is particularly crucial when determining the subcellular location of a cellular protein that is naturally present in low quantities.
7. Conversely, viral infection can result in high viremia, leading to the abundant production of viral proteins within cells. To simulate this scenario, each viral protein can be expressed over time using the full strength of the *nmt*1 promoter without thiamine. The impact of high viral protein expression levels on subcellular localization can then be documented and compared with low levels of expression [14].
8. For measuring potential co-localization of two proteins, it is advisable to use two different fluorescent protein tags with non-overlapping excitation and/or emission spectra. YFP and CFP are commonly employed for this purpose. While GFP is widely used, its excitation spectrum overlaps with both YFP and CFP. Therefore, GFP is not ideal for co-localization studies. However, since YFP cannot be excited under the excitation spectrum of the CFP filter, which has an excitation of 436/20 nm and emission of 480/40 nm, no YFP signal is detected under the CFP filter. Thus, it can be used to differentiate the YFP signal from GFP [14].
9. Other fluorescent microscopes equipped with suitable filter cubes can also be utilized to observe these fluorescent proteins.
10. It should be emphasized that utilizing fission yeast as a surrogate for virologic studies certainly has its limitations, as it may not fully represent the complex cellular interactions involving in a viral protein of interest. Therefore, subsequent validation of identified viral protein properties in the host cell and within the context of viral infection is imperative. Also, note that this protocol is tailored for investigating viral proteins in fission yeast. All molecular constructs described here are intended for use in fission yeast studies only. Consequently, the viral gene-of-interest identified within the fission yeast system must be cloned into a mammalian expression vector for translational studies in mammalian cells.
11. NEBuilder^®^ HiFi DNA Assembly offers a streamlined process for inserting one or multiple DNA fragments into the pYZ1N plasmid in a single step, ensuring proper orientation (Figure S1). For a single insertion, only the linearized pYZ1N and the PCR product of the viral gene are required. The forward primer of the viral gene (primer VG-F) should contain a 5′-end sequence overlapping by 20–30 bp with the *nmt* promoter of the pYZ1N plasmid and a 3′-end sequence overlapping by 20 bp with the viral gene start site (containing the ATG start codon). Similarly, the reverse primer of the viral gene (primer VG-R) should include a 5′-end sequence overlapping by 20–30 bp with the *nmt* terminator of the pYZ1N plasmid and a 3′-end sequence overlapping by 20 bp with the viral gene end site (including the stop codon).

## Figures and Tables

**Table 1:** Fission yeast strains and plasmids.

Strains/Plasmids	Genotype and Characteristics	Source or Reference
Fission yeast strains		
972	Wild type, *h^−^*; the original strain	[20]
975	Wild type, *h*^+^; isogenic to 972	
968	Wild type, *h*^90^; isogenic to 972	
SP223	Wild type, *h^−^*, *ade*6–216, *leu*1–32, *ura*4–294	Laboratory collection
**Plasmids**		
pYZx1N Vector Series		
pYZ1N	Fission yeast expression vector with an inducible *nmt*1 promoter and a *LEU*2 selectable marker; a derivative of pREP1; wild-type *nmt*1 promoter with a high level of expression	[11,13]
pYZ1N-bleMX6	Fission yeast expression vector with an inducible *nmt*1 promoter and a *bleMX*6 selectable marker; a derivative of pREP1; wild-type *nmt*1 promoter with a high level of expression	[21]
pYZ41N	Same as pYZ1N, but with an intermediate-strength *nmt*41 promoter; intermediate level of expression	[11,13]
pYZ81N	Same as pYZ1N, but with a low-strength *nmt*81 promoter; low level of expression	[11,13]
pYZ2N	Same as pYZ1N but with a *URA*4 selectable marker	[11,13]
pYZ3N-xFP Series		
pYZ3N-GFP	Same as pYZ1N but with a 5′ GFP fluorescent tag	[11]
pYZ3N-YFP	Same as pYZ1N but with a 5′ YFP fluorescent tag	Laboratory collection
pYZ3N-CFP	Same as pYZ1N but with a 5′ CFP fluorescent tag	Laboratory collection

**Note:** Fission yeast has three mating types, i.e., *h*^+^, *h*^−^, and *h*^90^. The *h*^+^ and *h*^−^ mating types are heterothallic, i.e., it needs an opposite mating type to mate. The *h*^90^ is homothallic, i.e., it can mate by itself.

## Data Availability

The data presented in this study are available on request from the corresponding author.

## Supplementary Materials

The following supporting information can be downloaded at: https://www.mdpi.com/article/10.3390/pathogens13070566/s1, Figure S1. Restriction map of fission yeast gene expression vector pYZ1N. Figure S2. Restriction map of fission yeast gene expression vector pYZ3N-GFP.

## References

[1] Hartwell L.H. (2004). Yeast and cancer. Biosci. Rep..

[2] Nasmyth K. (2001). A prize for proliferation. Cell.

[3] Nurse P. (2002). Cyclin dependent kinases and cell cycle control (nobel lecture). Chembiochem.

[4] Ray K. (2014). From fission to fusion: A perspective on the research that won the Nobel Prize in Physiology or Medicine, 2013. J. Biosci..

[5] Zhao R.Y. (2017). Yeast for virus research. Microb. Cell.

[6] Zhang J., Hom K., Zhang C., Nasr M., Gerzanich V., Zhang Y., Tang Q., Xue F., Simard J.M., Zhao R.Y. (2024). SARS-CoV-2 ORF3a Protein as a Therapeutic Target against COVID-19 and Long-Term Post-Infection Effects. Pathogens.

[7] Zhao Y., Elder R.T. (2000). Yeast perspectives on HIV-1 Vpr. Front. Biosci..

[8] Zhao R.Y., Elder R.T. (2005). Viral infections and cell cycle G2/M regulation. Cell Res..

[9] Andreola M.L., Litvak S. (2012). Yeast and the AIDS virus: The odd couple. J. Biomed. Biotechnol..

[10] Lista M.J., Voisset C., Contesse M.A., Friocourt G., Daskalogianni C., Bihel F., Fahraeus R., Blondel M. (2015). The long-lasting love affair between the budding yeast *Saccharomyces cerevisiae* and the Epstein-Barr virus. Biotechnol. J..

[11] Zhao Y., Elder R.T., Chen M., Cao J. (1998). Fission yeast expression vectors adapted for large scale cloning and GFP fusion with positive screening. BioTechniques.

[12] Li G., Zhao R.Y., Singleton T. (2018). Molecular Cloning and Characterization of Small Viral Genome in Fission Yeast. Methods in Molecular Biology.

[13] Maundrell K. (1990). nmt1 of fission yeast. A highly transcribed gene completely repressed by thiamine. J. Biol. Chem..

[14] Li G., Poulsen M., Fenyvuesvolgyi C., Yashiroda Y., Yoshida M., Simard J.M., Gallo R.C., Zhao R.Y. (2017). Characterization of cytopathic factors through genome-wide analysis of the Zika viral proteins in fission yeast. Proc. Natl. Acad. Sci. USA.

[15] Nkeze J., Li L., Benko Z., Li G., Zhao R.Y. (2015). Molecular characterization of HIV-1 genome in fission yeast *Schizosaccharomyces pombe*. Cell Biosci..

[16] Zhang J., Li Q., Cruz Cosme R.S., Gerzanich V., Tang Q., Simard J.M., Zhao R.Y. (2022). Genome-Wide Characterization of SARS-CoV-2 Cytopathogenic Proteins in the Search of Antiviral Targets. mBio.

[17] Jin H., Du Z., Zhang Y., Antal J., Xia Z., Wang Y., Gao Y., Zhao X., Han X., Cheng Y. (2020). A distinct class of plant and animal viral proteins that disrupt mitosis by directly interrupting the mitotic entry switch Wee1-Cdc25-Cdk1. Sci. Adv..

[18] Rallis C., Bahler J. (2016). Cell-based screens and phenomics with fission yeast. Crit. Rev. Biochem. Mol. Biol..

[19] Sahaya Glingston R., Yadav J., Rajpoot J., Joshi N., Nagotu S. (2021). Contribution of yeast models to virus research. Appl. Microbiol. Biotechnol..

[20] Moreno S., Klar A., Nurse P. (1991). Molecular genetic analysis of fission yeast *Schizosaccharomyces pombe*. Methods Enzymol..

[21] Benko Z., Zhao R.Y. (2011). Zeocin for selection of bleMX6 resistance in fission yeast. Biotechniques.

[22] Basi G., Schmid E., Maundrell K. (1993). TATA box mutations in *the Schizosaccharomyces pombe nmt*1 promoter affect transcription efficiency but not the transcription start point or thiamine repressibility. Gene.

[23] Forsburg S.L., Sherman D.A. (1997). General purpose tagging vectors for fission yeast. Gene.

[24] Siam R., Dolan W.P., Forsburg S.L. (2004). Choosing and using *Schizosaccharomyces pombe* plasmids. Methods.

[25] Forsburg S.L. (1993). Comparison of *Schizosaccharomyces pombe* expression systems. Nucleic Acids Res..

[26] Javerzat J.P., Cranston G., Allshire R.C. (1996). Fission yeast genes which disrupt mitotic chromosome segregation when overexpressed. Nucleic Acids Res..

[27] Benko Z., Elder R.T., Liang D., Zhao R.R. (2010). Fission yeast as a HTS platform for molecular probes of HIV-1 Vpr-induced cell death. Int. J. High Throughput Screen..

[28] Cormack B.P., Valdivia R.H., Falkow S. (1996). FACS-optimized mutants of the green fluorescent protein (GFP). Gene.

[29] Zhao Y., Lieberman H.B. (1995). *Schizosaccharomyces pombe*: A model for molecular studies of eukaryotic genes. DNA Cell Biol..

[30] Olsson I., Bjerling P. (2011). Advancing our understanding of functional genome organisation through studies in the fission yeast. Curr. Genet..

[31] Gibson D.G., Young L., Chuang R.Y., Venter J.C., Hutchison C.A., Smith H.O. (2009). Enzymatic assembly of DNA molecules up to several hundred kilobases. Nat. Methods.

[32] Benko Z., Zhang J., Zhao R.Y. (2019). Development of A Fission Yeast Cell-Based Platform for High Throughput Screening of HIV-1 Protease Inhibitors. Curr. HIV Res..

[33] Zhao Y., Cao J., O’Gorman M.R., Yu M., Yogev R. (1996). Effect of human immunodeficiency virus type 1 protein R (vpr) gene expression on basic cellular function of fission yeast *Schizosaccharomyces pombe*. J. Virol..

[34] Zhang J., Cruz-Cosme R., Zhang C., Liu D., Tang Q., Zhao R.Y. (2024). Endoplasmic reticulum-associated SARS-CoV-2 ORF3a elicits heightened cytopathic effects despite robust ER-associated degradation. mBio.

[35] Xu D.D., Du L.L. (2022). Fission Yeast Autophagy Machinery. Cells.

[36] Cruz-Cosme R., Zhang J., Liu D., Mahase V., Sallapalli B.T., Chang P., Zhang Y., Teng S., Zhao R.Y., Tang Q. (2022). A novel diG motif in ORF3a protein of SARS-CoV-2 for intracellular transport. Front. Cell Dev. Biol..

[37] Vida T.A., Emr S.D. (1995). A new vital stain for visualizing vacuolar membrane dynamics and endocytosis in yeast. J. Cell Biol..

[38] Chen M., Elder R.T., Yu M., O’Gorman M.G., Selig L., Benarous R., Yamamoto A., Zhao Y. (1999). Mutational analysis of Vpr-induced G2 arrest, nuclear localization, and cell death in fission yeast. J. Virol..

[39] Zhao Y., Yu M., Chen M., Elder R.T., Yamamoto A., Cao J. (1998). Pleiotropic Effects of HIV-1 Protein R (Vpr) on Morphogenesis and Cell Survival in Fission Yeast and Antagonism by Pentoxifylline. Virology.

[40] Forsburg S.L., Nurse P. (1991). Cell cycle regulation in the yeasts *Saccharomyces cerevisiae* and *Schizosaccharomyces pombe*. Annu. Rev. Cell Biol..

[41] Klionsky D.J., Abdelmohsen K., Abe A., Abedin M.J., Abeliovich H., Acevedo Arozena A., Adachi H., Adams C.M., Adams P.D., Adeli K. (2016). Guidelines for the use and interpretation of assays for monitoring autophagy (3rd edition). Autophagy.

[42] Kucsera J., Yarita K., Takeo K. (2000). Simple detection method for distinguishing dead and living yeast colonies. J. Microbiol. Methods.

[43] Benko Z., Elder R.T., Li G., Liang D., Zhao R.Y. (2016). HIV-1 Protease in the Fission Yeast *Schizosaccharomyces pombe*. PLoS ONE.

[44] Matsuyama A., Arai R., Yashiroda Y., Shirai A., Kamata A., Sekido S., Kobayashi Y., Hashimoto A., Hamamoto M., Hiraoka Y. (2006). ORFeome cloning and global analysis of protein localization in the fission yeast *Schizosaccharomyces pombe*. Nat. Biotechnol..

