# Supplementary figures and images for: Advanced Protocol for Molecular Characterization of Viral Genome in Fission Yeast (Schizosaccharomyces pombe)
Source: Pathogens. 2024 Jul 4;13(7):566. doi: 10.3390/pathogens13070566 (PMC11279667; doi:10.3390/pathogens13070566)

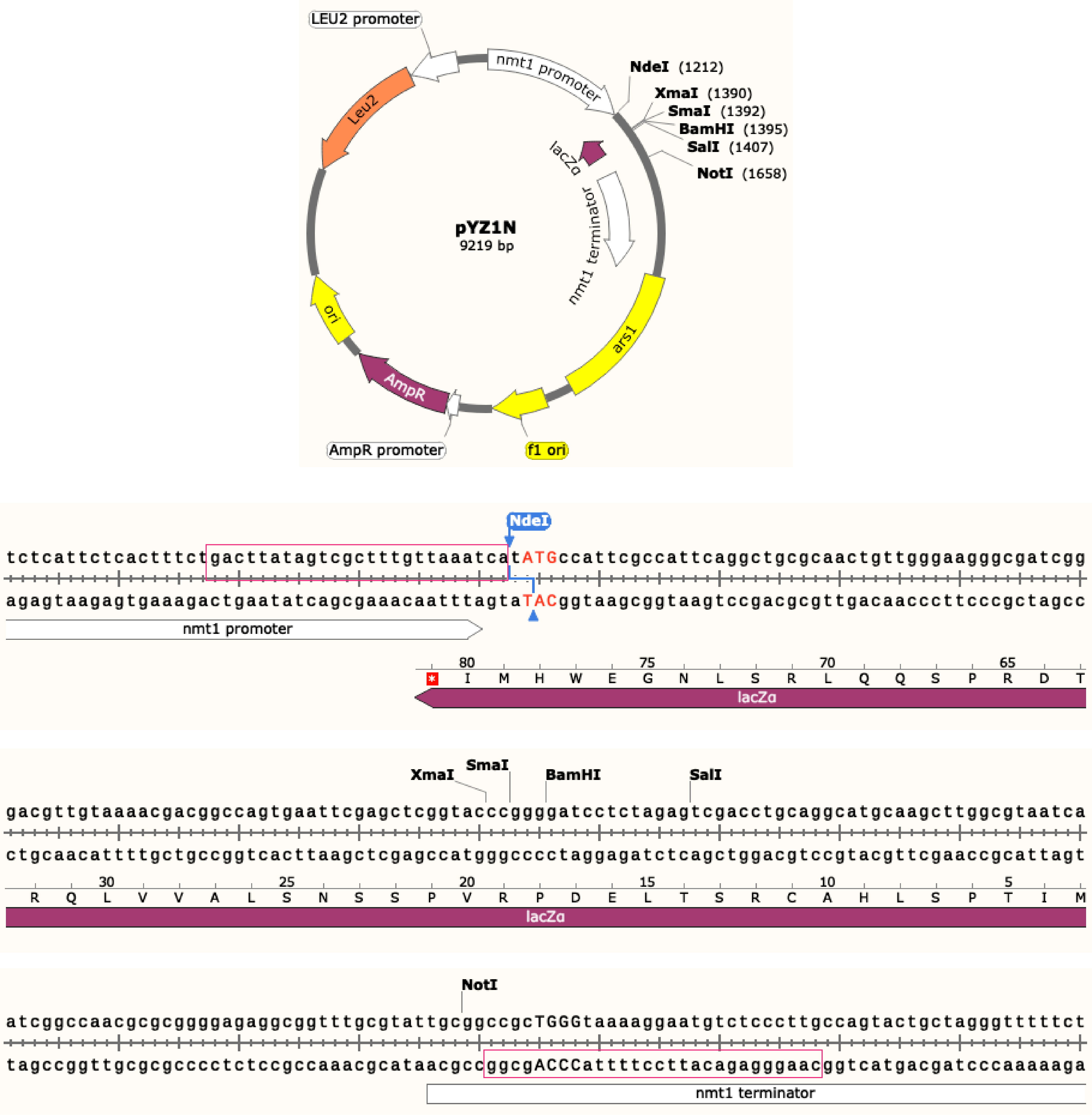

Supplement: Supplementary file 1 [file pathogens-13-00566-s001.zip › Figure S1.tif]

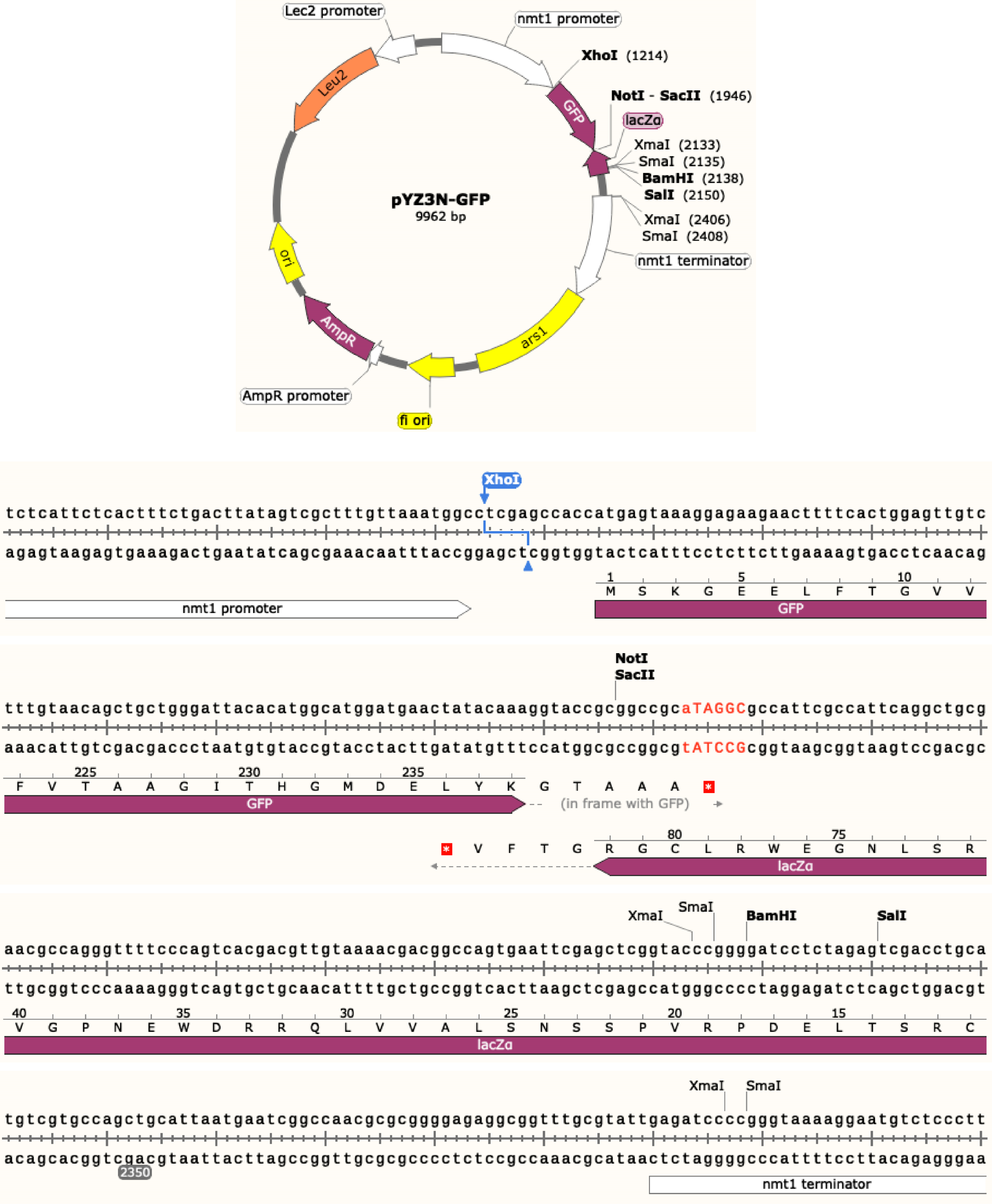

Supplement: Supplementary file 1 [file pathogens-13-00566-s001.zip › Figure S2.tif]
